# A twisted diagnosis of chest pain: the prominent role of coronary computed tomography

**DOI:** 10.1007/s12471-024-01889-1

**Published:** 2024-08-08

**Authors:** Catarina Amaral Marques, Cátia Oliveira, Ana Margarida Lebreiro, Mariana Vasconcelos, João Rebelo, Rui A. Rodrigues

**Affiliations:** São João Hospital Centre, Porto, Portugal

A 39-year-old man was admitted to hospital with sudden-onset chest pain at rest, which gradually disappeared. It progressed while walking and resolved at rest. Physical examination and initial diagnostic work-up were unremarkable [serial electrocardiogram, chest radiograph and echocardiogram (Fig. 1a; Videos S1 and S2, Electronic Supplementary Material)]. D‑dimer and troponin testing were negative. Coronary computed tomography angiography (CCTA) ruled out coronary artery disease (Fig. 1b–d) but unmasked the aetiology of the chest pain—epipericardial fat necrosis (EFN) (Fig. 1e–g; Videos S3 and S4, Electronic Supplementary Material). One month after discharge and a short course of anti-inflammatory therapy, the patient was re-evaluated, presenting no symptomatic recurrence and near-complete resolution of EFN (Fig. 1h).

**Fig. 1:**
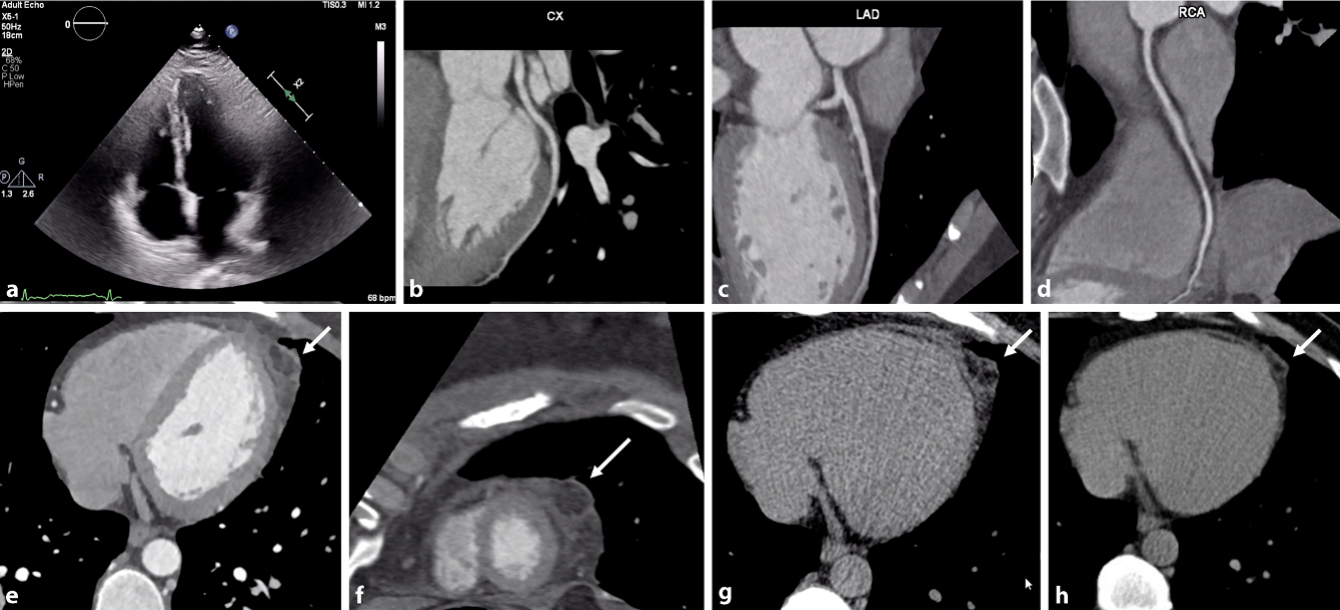
**a–h** Diagnostic work-up performed to clarify the aetiology of the chest pain. **a** Apical four-chamber image of the unremarkable transthoracic echocardiogram performed at admission. **b–d** Coronary computed tomography angiography (CCTA) images of the circumflex coronary artery (*CX*), left anterior descending coronary artery (*LAD*) and right coronary artery (*RCA*). **e–g** CCTA images showing a lesion (22 mm × 15 mm) located anterior to the left ventricle (*arrows*), suggestive of epipericardial fat necrosis (EFN); the less common CT pattern of EFN is shown here, comprising an ovoid fat mass with dense stranding. **h** Re-evaluation image showing almost complete resolution of the EFN (*arrow*)

This case highlights an uncommon and benign cause of chest pain [1] (typically mimicking life-threatening conditions), as well as atypical presentation and less common findings of EFN. The patient represents the classically described young male patient [2], although he did not present with pleuritic pain, the commonest presentation [3]. CCTA has a central role in the diagnosis of EFN.

Our work aims to raise awareness for this benign entity and, therefore, to avoid unnecessary and potentially deleterious interventions.

## Supplementary Information

Short axis and apical four-chamber views of the transthoracic echocardiogram performed at admission showing unremarkable findings.

Short axis and apical four-chamber views of the transthoracic echocardiogram performed at admission showing unremarkable findings.

Coronary computed tomography angiography (CCTA) showing normal coronary arteries and the lesion suggestive of epipericardial fat necrosis (EFN).

Coronary computed tomography angiography (CCTA) showing normal coronary arteries and the lesion suggestive of epipericardial fat necrosis (EFN).
